# Transition to adult mental health services for young people with Attention Deficit/Hyperactivity Disorder (ADHD): a qualitative analysis of their experiences

**DOI:** 10.1186/1471-244X-13-74

**Published:** 2013-03-05

**Authors:** Katie D Swift, Charlotte L Hall, Vic Marimuttu, Lucy Redstone, Kapil Sayal, Chris Hollis

**Affiliations:** 1 Child and Adolescent Mental Health Services, Nottinghamshire Healthcare NHS Trust, Nottingham, United Kingdom; 2 CLAHRC, the University of Nottingham, Nottingham, United Kingdom; 3 Developmental Psychiatry, University of Nottingham, Queens Medical Centre, Nottingham, UK

**Keywords:** ADHD, Transition, CAMHS, AMHS, Thematic analysis

## Abstract

**Background:**

There is little research on the process of transition between child and adolescent mental health services (CAMHS) and adult mental health services (AMHS). More recently, there is growing recognition that Attention Deficit/Hyperactivity Disorder (ADHD) may persist into adulthood requiring services beyond age 18. However, despite National Institute for Health and Clinical Excellence (NICE) Guidance which recommends specialist services for adults with ADHD, there is currently a lack of such services in the UK. The aim of the current study is to explore the experiences of young people with ADHD during transition from CAMHS to AMHS.

**Method:**

Semi-structured qualitative interviews with ADHD patients accessing CAMHS clinics in Nottinghamshire were analysed using thematic analysis.

**Results:**

Ten semi-structured interviews were transcribed and analysed. We found that patients’ relationships with their clinician were a key factor in both their reported experience of CAMHS and the transition process. Perceived responsibility of care was also pivotal in how the transition process was viewed. Nature and severity of problems and patients expectations of adult services were also contributing factors in the transition process. The need for continued parental support was openly accepted and thought to be required by the majority of young people with ADHD during transition.

**Conclusions:**

Timely preparation, joint working, good clinician relationships and parental support serve to facilitate the process of transition for young people with ADHD. Nature and severity of problems are perceived to impede or facilitate transition, with predominantly more ‘complex presentations’ with associated mental health problems more familiar to AMHS (e.g. self-harm, depression) making for smoother transitions to adult services. Transitions to AMHS were more difficult when ADHD was viewed as the main or sole clinical problem. Further exploration of young people’s experiences of transition and their engagement with and experience of adult services is required to provide an overall picture of facilitators to successful transition and integration into adult services.

## Background

Young people’s transition from children and young people’s services to adult health care services has received much attention in the field of physical health [1-4]. Comparatively, little attention has addressed the transition process in mental health services. Transition between child and adult health services is described as “a purposeful planned process that addresses the medical, psychosocial and educational/vocational needs of adolescents and young adults with chronic physical and medical conditions as they move from child-centred to adult-oriented health care systems” [5]. Young people with mental health concerns represent a particularly vulnerable group [6,7], in which a smooth transition is crucial to ensure that the young person’s needs are continued to be met. Of particular interest are young people with Attention Deficit/Hyperactivity Disorder (ADHD). Recently, there has been growing recognition that ADHD is a lifespan neurodevelopmental disorder that commonly persists into adulthood, with approximately 2-3% of adults meeting the criteria for ADHD [8]. The continuation of ADHD symptoms into adulthood can have a significant impact, with adults with ADHD having more driving accidents, higher divorce rates, greater risk of substance misuse, and changing job more frequently than adults without ADHD [8-10]. It is therefore crucial for young people diagnosed with ADHD to be able to access continuing mental health support into adulthood as advocated by NICE [11].

Despite this recognised need for continuing support, there is little published research on the transition from child and adolescent mental health services (CAMHS) to adult mental health services (AMHS), and unlike areas of physical health care (e.g., Cystic Fibrosis [2]) there is no accepted universal model to aid the transition of young people with ADHD to adult services. Although, some models of transition are evident in the literature (e.g., the ADHD transition clinic, Rotherham, South Yorkshire, [12]) the process is often turbulent and in some cases impossible [13].

A few quantitative studies have highlighted some of the issues arising in relation to transition into adult mental health services (AMHS). A recent audit of transitional care for ADHD patients showed that whilst 104 patients were eligible for transition,73% of patients were either discharged or lost in follow-up [14]. These findings support an earlier mixed methods study [15] which also found a low rate of successful transitions, specifically noting pronounced difficulties for young people with neurodevelopmental disorders, such as ADHD. The study found less than 5% of CAMHS cases achieved optimum transition. This study described ‘optimum transition’ as having a number of important stages, such as good information transfer across services, joint working, and continuity of care following transition [15]. Although quantitative studies provide useful figures for successful and unsuccessful transition, qualitative research can provide us with a unique insight into the often turbulent journey from CAMHS to AMHS and how this journey is experienced by young people and their families.

Qualitative studies exploring transition have typically focused on physical health conditions. Soanes and Timmons [16] examined the needs and attitudes of young people with chronic illness in transition, and identified five key themes; these included comfort and familiarity with the new clinician, concerns of potential over- formality in adult services, the need for a graduated and adequate preparation process, the need for flexibility, and finally concerns surrounding support. In another study, Kirk [17] explored the experiences of transition for young people with complex healthcare needs using in-depth qualitative interviews and analysis based on grounded theory principles. They noted that whilst the transition to adult services was occurring, often this was concurrent with other life changes. This is consistent with the need to view adolescence as a ‘life stage’ [18] and it is therefore prudent to remember that whilst service transitions are occurring, biological, social and psychological changes should also be considered. Furthermore, Kirk found that parents did not feel wholly informed about the process, which was mirrored by young people who expressed a lack of preparation, often experiencing an abrupt move from one service to another [17].

In a recent study, Van Staa et al. [19] used semi structured interviews to map the experience of transition to adult care for young people with chronic conditions, such as diabetes and haemophilia. They identified four key themes; these included leaving paediatric care being a logical step, cultural gaps between child and adult services, preparation of young people and parents/carers for the differences between child and adult services, including increased self-management of care and lastly collaboration and links were also viewed to be important. The authors make recommendations to improve patients’ experience of transition including preparing the young person and family early, strengthening adolescent independence without undermining parental involvement and gaining new trusting relationships. They suggest that transition should be a process of ‘responding and bonding’ which views parents as partners in a purposeful and planned process.

One of the few studies to investigate transitioning between mental health services conducted a thematic analysis on interviews with 11 service users approaching the transition stage [15]. They found that informal and gradual preparation, meetings to discuss transfer, joint-working between CAMHS and AMHS and consistency in key workers all played a vital role in the transition period. This study identified a cohort of CAMHS service users transitioning to AMHS. Interestingly, the quantitative part of the study highlighted patients with neurodevelopmental disorders (such as ADHD) as one of the most likely to not transition successfully to AMHS [15].

From the small, but growing literature it would appear that transition can be a turbulent journey, which is heightened for those with neurodevelopmental disorders and often results in discharge from a child service rather than entry into an adult service. Often appropriate adult services are not available and where they are, difficulties such as thresholds (e.g., AMHS acceptance criteria) [20], lack of clinician expertise in disorders typically presenting in childhood [21] and care cultures [22] (e.g. systemic vs. person centred) continue to hinder continuity of care. Consequently, little evidence exists in relation to what young people with ADHD want and need from an adult service. The aim of the current study is to qualitatively explore the experiences of young people with ADHD during transition from CAMHS to AMHS.

## Method

### Participants

Participants were identified though the young person’s CAMHS clinician. All CAMHS clinicians (which included psychiatrists and psychologists) across Nottinghamshire were approached by the lead researcher (KDS) either at the clinic they worked in or at their monthly professional meeting to help with the recruitment of young people aged 17-years and over with a diagnosis of ADHD or psychotic illness. Clinicians were required to either send a study pack to the young person, which included information sheets, consent forms and questionnaires or to gain permission from the young person and their family to pass on their details to a researcher. Unfortunately due to services not having a single database which records patient diagnosis and some clinics using temporary members of staff who were not fully familiar with their caseload, we were unable to establish the total number of young people meeting the eligibility criteria accessing CAMHS. Service-users who returned a completed questionnaire pack and reply-slip were invited to take part in the interview. Of the 15 ADHD participants recruited to the study, two participants did not complete the reply slip and therefore could not be contacted regarding the interview. A further three participants declined to be interviewed due to time constraints.

Thus, overall only ten interviews were carried out of which five participants were interviewed with their biological parent only. For two interviews, more than one family member was present. The remaining three interviews were carried out with only the young person present. Of the 10 participants, three had transitioned to adult mental health services at the time of interview, however upon recruitment to the study were all pre-transition. Recruitment spanned one year (September 2010-September 2011), and no further participants were recruited after this point. The study was approved by Derby Research Ethics Committee and Trent CLRN. Participant demographic information is presented in Table 1.

**Table 1:** Participant demographics, knowledge of transition and transition stage

**Participant number**	**Age**	**Gender**	**Ethnicity**	**Diagnosis**	**Co-morbidities**	**Treatment**	Knowledge of transition*	**Transition stage at time of interview∞**
P1	17 yrs 4 mths	Female	White British	ADHD	Self Harm	Stimulant Medication	No	Pre Transition
P2	17 yrs 4 mths	Male	White British	ADHD	NA	Stimulant Medication	Yes	Pre Transition
P3	18 yrs 3 mths	Male	White British	ADHD	Tourettes including Head Tics and Aggression	Stimulant Medication, Non Stimulant ADHD Medication & Antipsychotics	No	Pre Transition
P4	17 yrs 7 mths	Male	White British	ADHD	Communication	Stimulant Medication	Yes	Pre Transition
P5	18 yrs	Male	White British	ADHD	NA	Stimulant Medication	Yes (4 months prior to interview)	Transitioned (Multiple previous failed attempts)
P6	18 yrs 3 mths	Female	White British	ADHD	Autism, Self Harm, Depression	Melatonin, Olanzapine & Concerta, Plus CBT & Client Centred Approaches	Yes	Transitioned
P7	18 yrs 6 mths	Male	White British	ADHD	Autism (Aspergers), Epilepsy, Dyslexia, Aggression	Stimulant Medication, Antipsychotics and Antidepressants	Yes (1 month before 18^th^ Birthday)	In transition
P8	17 yrs 10mths	Male	White British	ADHD	Autism	Non stimulant ADHD medication, plus family therapy work and parenting support and advice	Yes	Transitioned (3mths in Adult then discharge to Adult Social Care)
P9	17 yrs 1 mth	Male	White British	ADHD	Low self esteem (Previously: Self Harm & Depression)	Solution focused and motivational work	Yes	To be discharged
P10	18 yrs 4 mths	Male	White British	ADHD	Depression	Anti-depressant medication	NA	Discharged – Now requiring re entry into services

*At time of Interview/When Mentioned ∞NB. On recruitment (postal questionnaire) all participants were pre-transition.

#### Procedure

Semi structured interviews were undertaken as part of the Transition to Adult Mental Health Services (TRAMS) project in Nottingham. The project is a mixed method prospective 12 month study, which uses both postal questionnaires and semi structured qualitative interviews to assess young people’s experience of transition from child to adult mental health services. The present study reports findings from the initial baseline qualitative interviews.

The interviews were undertaken by one of two of the authors (KDS or VM) and recorded on a Dictaphone. The interviews were semi structured consisting of five set questions (see Table 2), which asked about the young person/family’s experience of CAMHS and transition to adult mental health services. The use of a semi structured interview format allowed the researchers flexibility to ask additional questions based on the interviewees responses.

**Table 2:** Semi-structured interview questions

**Question Number**	**Questions**
**1**	Could you tell me the story about how you first came to see someone at CAMHS?
**2**	Could you tell me about your experiences of using CAMHS?
**3**	Are you aware of any plans for your care to be transferred from CAMHS to AMHS when you turn 18 years old?
**4**	Can you tell me about what you feel and think about this?
**5**	What do you think is the best way to help prepare you for this transfer?

The interviews all took part in the family home, and were arranged at a time that was most convenient for the young person, their family and key worker where applicable. It was the young person and their family’s decision as to who participated in the interview and no restrictions were placed on the number of family members present. Consent was gained prior to the interview commencing and consent forms where completed for all participants (young person & family members) taking part in the interview.

#### Data analysis

Audio recordings were anonymised and transcribed verbatim. Analysis of transcripts was carried out by two of the study researchers (VM & KDS) and one independent researcher (CH) under supervision of a qualified Clinical Psychologist (LR) with experience in Thematic Analysis. The analysis followed the process recommended by Braun and Clarke [23] shown in Table 3. Reviewers undertook an inductive approach, therefore the analysis was data driven and did not aim to fit into a pre-existing coding frame, or the researcher’s analytic preconceptions. Inductive approaches strengths lie in the allowance for exploratory (as opposed to confirmatory) analysis of the data. Using this approach, researchers get to know the data through re-reading it numerous times before any analysis begins. This method is suitable for team research and its rigor is improved by interpretation being supported by the data. Conversely, interpretation can be a weakness of this method and reliability can be compromised, therefore inter-coder agreements are required. In this study each analyser took the same approach to the data. A lead analyser (KDS) was appointed to co-ordinate the process of addressing theme consistency. Overall, themes were largely consistent between coders, whereby contradictory coding was apparent, discussions with all coders and triangulation with the full data set was undertaken to achieve consensus. The researchers’ epistemology was one of an essentialist/realist paradigm, which views the meanings, experiences and realities of participant’s experiences of transitions as assuming a unidirectional relationship between meaning, experience and language [23]. This sought to understand the experiences of transition through the words of the participants, placing less emphasis on the researcher co -creating meaning.

**Table 3:** Phases of thematic analysis taken from Braun & Clarke (2006)

**Phase No.**	**Phase Description**
1	Familiarising yourself with your data: Transcribing data (if necessary), reading and rereading the data, noting down initial ideas.
2	Generating initial codes: Coding interesting features of the data in a systematic fashion across the entire data set, collating data relevant to each code
3	Searching for themes: Collating codes into potential themes, gathering all data relevant to each potential theme
4	Reviewing themes: Checking in the themes work in relation to the coded extracts (Level 1) and the entire data set (Level 2), generating a thematic ‘map’ of the analysis.
5	Defining and naming themes: Ongoing analysis to refine the specifics of each theme, and the overall story the analysis tells; generating clear definitions and names for each theme.

## Results

Approximately 81 young people with ADHD were identified through 11 CAMHS clinicians. Of these 81, some no longer had a clinical diagnosis of ADHD but remained in the service as they were receiving treatment for other conditions (e.g. depression). Furthermore, some cases were already progressing through transition or had been discharged from the service. Other reasons for attrition included not responding to questionnaire packs or attempts to contact.

We report on themes extracted from the 10 (12% of the sampling frame) ADHD participants’ interviews.

### Themes

Four key themes emerged of young people and parents/carers experiences of CAMHS and transition; clinician qualities and relationship, responsibility of care, nature and severity of problems, and expectations of AMHS.

### Clinician qualities and relationships

Almost all of the young people and parents across our sample expressed that clinician qualities and relationships were a pivotal part of their experience of both CAMHS and the transition process.

Their views pertained to the individual qualities of the clinician, which in turn affected the relationship between patient and clinician and the patient’s engagement with the service. It transpired that clinician qualities were very important and often imperative in terms of how patients viewed the service, for example a ‘good clinician’ often resulted in a positive view of the overall service. Particular qualities which seemed to be repeatedly acknowledged included clinicians being ‘nice’, ‘understanding’, ‘supportive and informative’ ‘non- judgemental’ and ‘good listeners’. Listening was a particularly important characteristic and received a lot of attention throughout the majority of the transcripts.

“It was quite good ‘cos It was more it wasn't you didn't sit down and it want like a face to face talking it was like a friend talking asking you a few questions it wasn't really that direct” [P10]

“She listened to X and she never judged and she came up with good practical ideas that were achievable and realistic, whereas the one before was just, she didn’t listen” [P6]

Interestingly, several of the participants had experiences with more than one clinician and were able to reflect and compare their experiences of individual clinicians’ qualities and conduct. Inconsistencies including gaps in the service and changes of clinician were common viewed negatively. Those that had experienced contact with more than one clinician reported differences in their relationship with the clinician. It was evident that disparities between clinician’s qualities and conduct led to differences in satisfaction with the service provided. Clinicians who were perceived as not listening or not providing a useful service were viewed negatively and participants reported disengagement from the service or the request for a new clinician as a result.

“no hit and miss, oh with that person yes she has got loads achieved, but over the years it’s been hit and miss you’re lucky as to who you get as to how good a service you get” [P6]

Another quality that was viewed positively was the clinician’s willingness to ‘go the extra mile’. Some participants reported that they felt their clinician went above and beyond their duty, whilst others were dissatisfied and felt ‘dumped’ by their clinician and the service. Participants who reported clinician’s tenacity were particularly encouraging about their experience of CAMHS, and also their transition. Furthermore, parents and young people typically viewed the clinician’s ability to provide pace and flexibility to the process of transition positively. From our analysis, those participants whose clinician had appeared to work hard in the transition period reported a less turbulent transition process despite in some cases there being other difficulties, such as not meeting the threshold for adult services.

This was well illustrated in the case of participant 5, who had multiple failed transitions, and whose clinician provided support and re-assurance that they would do everything possible to ensure a successful transition to provide continued care.

“I always thought he was one of those people who always used to work hard for me, I don’t know about other people if they had different ones and different people. If I’d had to keep having different people every year then I couldn’t have coped with that because I would’ve preferred a person I can trust and talk to” [P7]

Similarly, this quote also highlights an important issue that was raised by many participants regarding consistency, which will be described next under ‘responsibility for care’.

#### Responsibility for care

An interesting theme emerged with regard to who is responsible for care and ensuring continuity of care. It emerged that the young people in our sample did not typically have sole responsibility for their care and were often reliant on family members for support. Support from parents was often of a practical nature and pertained to providing help with attendance at clinic appointments and taking medication. This inability to plan for appointments and reduced independence may be due to immaturity and reduced executive functioning skills associated with ADHD [24].

“I don’t know ‘cos I can see where they’re coming from ‘cos he’s an adult, but he’s an adult with something wrong with him, and that and they know he won’t go out the house and won’t do certain things on his own yet he’s got to go all the way up there and that’s the point he won’t do that” [P8]

Some interviews touched upon the difference in care culture between child and adult services and how this may impede their access to services due to adult services employing a more patient- centred approach and typically requiring more autonomy from the young person.

The clinician was perceived by participants as having responsibility for the transition process, and it arose that preparation for the process was not always provided, which resulted in some participants feeling thwarted and concerned as to how their needs would be met. Others, however, had a positive experience whereby the transition process was perceived as positive and demonstrated how the clinician has a responsibility to their patients to ensure that they are informed and are part of the process as this seemed to promote optimism regarding transition and during transition. Participants provided suggestions regarding how they could be prepared. These included being introduced by their current clinician to the clinician that would be taking over their care or seeing a photograph of them, being provided with written documentation regarding the process, including what will happen and where and when everything will take place. Furthermore, patients and families hoped they would see one clinician consistently in the new service. Patients and families wanted to feel prepared for the process, and therefore did not want to be ‘in the dark’ or feel unprepared. Orientation to adult services has been previously noted to be an important step in the transition process [25]. One participant brought to light the concept of service responsibility. The young person stressed that despite symptom severity a service should be available, thus further highlighting the idea of responsibility for continuity of care.

“I think that there should always be, like if you finish with a child, there always should be someone on the end of that to pick you up always, even if you’ve got less of ADHD than what I have, you should always, there should always be a solution at the end, even if that person wants you to come off the tablets and you have no choice, at least you’ve got somewhere, somewhere to help you,” [P5]

As previously noted, some participants had experience of contact with multiple clinicians. In light of this, a theme emerged regarding the services’ responsibility to provide a consistent and seamless service during both the patients’ time in CAMHS and during transition. Whilst it is likely and acceptable that clinicians will move posts, a clear handover could be easily implemented to avoid patients feeling that they are being left to begin the process of forming relationships and providing information again. Some patients experienced good continuity of care, through having one clinician throughout the majority of their time with CAMHS; however others found the service to be much more turbulent, with gaps in provision and changes of clinician, which they found disruptive. Furthermore, participants viewed transition as unsettling due to them having to change from one service to another and potentially not having a service to go to. In addition, some did not accept or understand why this change had to take place purely based on their age.

“I don't see what age has got to do with who you’re seeing and where you see ‘em. Right, we’re used to coming here, but now we’ve got to change and go somewhere else, so that’s a bit annoying” [P2]

Of the participants interviewed many did not see transition as a logical step and instead was viewed it as an unnecessary process, which resulted in participants feeling as though the service did not care or that they were being ‘dumped’ by the service. Furthermore, our findings support that young people do not feel age is an appropriate indicator and were perplexed as to why their age should affect their continuity of care.

One participant gave insight into how they would feel if they were not accepted by adult services.

“So I wasn’t really, erm, worried, but if I was someone else who never got a place, then I would understand that, that you’ve been left out and no-one cares and somebody should actually do something” [P5]

This participant believed that not being accepted by adult services would mean that ‘no one’ cared about them. Therefore, the ability of clinicians and services to provide accurate information to patients, specifically those with ADHD for whom adult services are not readily available is paramount. Such information may reduce the potential for patients to feel let down by services or that services are not ‘responsible’ for them.

### Nature and severity of problems

The severity of participants’ problems seemed important in relation to whether they would be accepted by adult services. Service thresholds have been noted previously as a barrier to transition [15]. One parent commented on how the young person would not remain in adult services if they ‘only’ had ADHD and autism, yet felt they would have access to the service if they had mental health difficulties, such as self harming.

“Well I’ve known for a while, but it’s like they was saying he would be under X Hospital to get the help, I thought it would be all the time, which it’s not, he just went for one appointment, to see how he was cos of course they think he, he’s got autism/ADHD but he’s not self harming or anything so he can go to his own doctor if he’s got no problems” [P8]

A similar comment was made by a young person regarding ADHD severity. They expressed that young people with mild to moderate ADHD should always have care provision and that choices should be available.

### Expectations of adult service

Young people and their parents/carers expressed concerns about transition to an adult service. In particular, parents expressed the desire for their child to receive a consistent service and for them to be included in consultations regarding their child’s care.

“When she gets to 18 is there gonna be somebody there that can talk to us and talk to her? Is it gonna happen because we don’t know. We just don’t know. And it worries you” [P1]

We found that families held high expectations of AMHS services, and in many cases these expectations are unlikely to be met, for example some parents expected adult mental health services would be on hand to help with housing, whereas this service would more likely be provided by adult social care.

“I hope that it can be the same person there for him from when he starts there and erm, and I just hope that it will be able to help him to get his own place eventually, not straight away, but eventually and help him to learn to get up in the morning to go to work and you know” [P4]

It is therefore imperative that CAMHS clinicians are mindful not to provide unrealistic expectations to patients regarding what they will receive or can expect from an adult service. In two cases, CAMHS clinicians had expressed to young people that AMHS would be the same as the service they received in CAMHS. One participant confirmed this and reported their experience of AMHS as the same as they had received in CAMHS. For parents, concerns typically focussed on access to a professional who could provide support and someone to talk to for both themselves and the young person. For young people, concerns centred around not only having access to treatment, but also concerns regarding the building they would have to go to, the format sessions would take, the other people they would meet, and forming a relationship with a new clinician.

“Yeah, will there be like people with the same disability, or people with like schizophrenia or any other serious illnesses - Yeah, and will it be a one-on-one as well ” [P2].

In summary, the issues highlighted in terms of expectations relate back to the clinicians qualities and their relationship with their client. It is the responsibility of both the clinician and the service to provide timely preparation for the prospect of transitioning to another service, and likewise important that the differences between these services and the type of care they will receive be thoroughly discussed. The provision of such information, providing consistency and helping to facilitate a new relationship with the adult service clinician appear paramount for the transition process to be viewed positively.

## Discussion & recommendations

Whilst transition between mental health services has been qualitatively explored [26], to date we believe this is the first study to qualitatively investigate the experiences of transition between mental health services for young people with ADHD. In the health literature, the issue of transition between child and adult services has been well discussed; however, the process in mental health services has been comparatively under-investigated. In this study, we sought to explicitly elicit the views and experiences of young people approaching or undergoing transition from CAMHS to AMHS. We found that patients’ relationships with their clinician were a key factor in both their reported experience of CAMHS and the transition process. The perception of perceived responsibility of care was also pivotal in how the transition process was viewed. The nature and severity of problems and patients expectations of adult services were also contributing factors in the transition process.

Young people and their carers/parents relationship with their clinician was perhaps the most poignant theme to emerge from our study. In line with previous research [18,27] it appeared that the patients’ experience of their interactions with their clinician and having a trusting relationship with them was an important factor to their satisfaction with CAMHS. Of particular importance was the need for patients to feel that they were being listened to. Price et al. [18] have suggested that communication should be a pivotal part of training for professionals delivering healthcare transition services so that they are able to engage young people as patients. Although this concept has been documented previously [28], it is perhaps particularly prudent in the cases of young adolescents, to allow them to feel respected and valued as young adults. It is possible that young people who feel that they are being listened to and have input in to their care feel more enabled to deal with their condition [18]. As such, it is possible that these cases may have better transition into adult services. Furthermore, those individuals with a positive experience of CAMHS may be more willing to engage with adult services once transitioned.

The transition to adult services brought up an interesting debate regarding responsibility of care. From our sample, it was evident that parental input was still desired or required in some cases. There was no evidence of young people not wishing to have parental support, supporting previous findings from physical health care literature that parents are still needed by their children in adult health services [29,30]. Some cases even suggested that they would not be willing to attend services without their parents present. This is somewhat contrary to Singh et al. [15] who observed that young people preferred not to have their parents involved in their care, although the parents still wanted to play an active role in their child’s contact with adult services, which we also observed despite parents openly acknowledging their child achieving adulthood. It is possible that post–transition our sample may also report the same preference and given that we often interviewed both the parent and young person together this may have influenced the young person’s voice. Alternatively, it may be that parental involvement is crucial in the early stages of transition to adult care. ADHD symptoms manifest in childhood, which often leads to an extended period of contact with CAMHS, this often means that parents are involved with their child’s care and support from an early age, which may explain the reliance on parents in these cases. It is therefore recommended that adult services adopt a flexible approach to parental involvement based upon patient need and preference.

There was also evidence that young people and parents saw their clinician as being responsible for the success of their transfer. Similar to the findings of Singh et al. [15], some of our sample had received sessions of ‘joint working’ between CAMHS and AMHS clinicians or felt that their clinician had prepared them for transition in to adult services. However, some participants reported feeling let down and ill-prepared to transition. Cases where young people felt their CAMHS clinician had prepared them for transition typically had a more positive view of both CAMHS and AMHS. Furthermore, clinicians which were perceived to work hard for their client viewed positively. This is echoed by the results of Soanes et al. [16] who suggest that adolescents be ‘steadily ‘prepared for transition. Similarly, Singh et al. [15] also found that flexibility and persistence of clinicians fostered better patient engagement. In addition the importance of preparation is stressed in both the NICE guidelines for ADHD (CG72) [11] and Department of Health documentation, “Transition: getting it right for young people” [5] which suggests full information should be provided to the young person about adult services and they should be involved in the planning process, which should begin as early as possible [5,11]. Tuchman et al. [25] suggest that earlier discussions, opportunities to meet new healthcare providers and visits to adult venues may aid the process of transition. Joint working has been previously documented as a potential facilitator to transition [15,16] and is advocated by NICE [11].

Consistent with the findings of Singh et al. [15] we also found that patients with more severe mental health issues were more likely to transition to AMHS than those with milder or less complex problems. It is possible that this reflects a severity threshold applied by AMHS or perhaps limited experience and expertise in childhood neurodevelopmental conditions, especially ADHD for which there is limited training for many healthcare professionals [13,28]. We found that parents perceived the nature and severity of symptoms as factors which could impede or facilitate transition. Thus it is recommended that further training in ADHD symptoms and difficulties or the provision of specialist consultation be advocated for AMHS services.

The importance of managing expectations and concerns surrounding adult services was also observed. Parents and children often had an unrealistic expectations as to what AMHS could offer, or simply did not know what to expect. This mirrors the findings of Wright [31] who found that parents and young people had elevated expectations as to which services would be provided in AMHS. This further highlights the noted differences between the services available in CAMHS and AMHS. This lack of knowledge tended to lead to concerns and fears surrounding transition. One participant expressed their concerns regarding their knowledge that an equivalent adult service may not be available and how their needs for ADHD medication prescribing and monitoring would be met, mirroring the findings of Kirk [17], whilst some young people adopted a ‘wait and see attitude’ as found by Singh et al. [19]. Whilst previous research has highlighted that young people and parents saw the transition to adult services as a logical step [19] this was not reflected in the participants we interviewed and instead transition was viewed as unnecessary and resulted in participants feeling as though the service did not care or that they were being ‘dumped’ by the service. Furthermore, transition based on age has been described as a bureaucratic barrier [20] which has been highlighted as a potentially insufficient indicator of readiness to transition [16]. Some participants did not see the need to transition and saw the potential transfer to an adult service as an inconvenience and annoying, which raises questions regarding transition appropriateness or readiness [19] and further supports the importance of preparation and expectation management. Of the participants who had already been transitioned, one participant noted no noticeable differences between CAMHS and AMHS. This may have been due to the service they received in CAMHS being predominantly medication monitoring and prescribing. However, experiences may differ whereby other treatments are required. For example, AMHS may not provide family therapy, a service which may be received in CAMHS. The Social Care Institute for Excellence (guideline 44) advocates clinicians having knowledge of how each other’s services operate in order to provide co-ordinated and joined up services [6]. It has been noted that there is often a lack of knowledge by both CAMHS and AMHS regarding service structures and available pathways [7], such lack of knowledge may hinder transition if clinicians are not aware as to what adult services can provide and what services are available.

### Limitations and strengths

Our study is limited by its small sample size. Although, unlike quantitative studies, qualitative studies are not required to achieve a certain sample size per se, the limited numbers require caution when generalizing to the wider population. Furthermore, our study was only conducted within one healthcare organisation and therefore comparison with other services whereby different ways of working may be in operation would be of benefit and may reveal contrasting results. Furthermore, not all young people with ADHD are seen in CAMHS. Originally the study hoped to also recruit from Pediatric services which should have likely increased the number of eligible young people with ADHD, however difficulties with using the same study materials as those used in CAMHS and obtaining ethical approval for conducting the study in additional service organisations meant this could not be achieved. Evidence from such studies is paramount if we are to develop appropriate evidence based protocols and ways of working for transition.

Some young people were interviewed with their family members; this may have been a weakness as often parents dominated the interviews and young people spoke through them. However, some young people may have spoken more due to their parent being present. Interestingly, those interviewed without their parents present were perceived by the interviewers to be more independent and expressed their independence from parental support in the interviews. In contrast, those interviewed with their family members appeared more heavily reliant on parental support.

Although some of the participants had recently transitioned to adult services, they were in the early stages of contact with AMHS. We found that in some cases our interviews provided the first introduction to transition, which was also experienced by Soanes et al. [16]. Our study was designed to observe the entire process of transition from 17 to 18 years of age. NICE guidelines [11] state that the transition process should start at 17.5 years and, as such, our findings regarding a lack of preparation for transition may reflect the fact that some participants would not be expected to have any knowledge regarding transition at that time. However, one participant at 18 years and 3 months of age had no knowledge of transition. Furthermore, whilst one participant under the age of 17.5 years did not have knowledge of transition, a further participant under 17.5 years was aware of the potential to be transitioned or discharged showing that the process is not uniform.

As this study is an analysis of baseline data and participants are at differing transition stages, further caution must be exercised in relation to the conclusions drawn as they may only be reflective of this point in time and the 12 month follow-up interviews may provide further insight into the overall experience of their journey from CAMHS to AMHS.

With regard to participating clinicians, we found that willingness to participate in the project for some clinicians was limited, resulting in few referrals to the study. In total, we contacted 24 clinicians, of whom 11 responded with a patient list identifying potential cases. In total, this resulted in 81 cases. Hence the 10 cases that agreed to participate in the study represent approximately 12% of the possible cases available. These 10 cases came from 7 clinicians (6 psychiatrists and 1 psychologist). Whilst we realize that the study demanded extra work from clinicians in order to facilitate recruitment, the clinician’s availability and motivation to aid the project recruitment may have been a further limitation of the study. In turn this may have influenced the findings as referring clinicians may have had a vested interest in transition. Furthermore, this affects the generalisability of the study as does the geographical area in which this study was set. Therefore our sample may not be representative of young people with ADHD in transition in other areas in the UK. In addition, the experience of transition for young people with ADHD is likely to be different in areas where dedicated transition policies and procedures are available. However, despite such policies our findings demonstrated that the clinicians themselves are pertinent to the process and the young person’s experience.

It is pertinent to remember that whilst the information collected from participants is invaluable, these are still individual perceptions and may not accurately reflect the process of transition or conduct of clinicians and services involved. In addition, triangulation of information, with for example, case notes may have enabled some verification of our findings. Whilst our sample was small, the presence of recurring themes and their support of previous research throughout the transcripts provide some reassurance regarding theme validity and therefore affords a degree of accuracy as to how ADHD patients are currently experiencing CAMHS and the transition process. Although some commonalities with previous research have been noted, generalization of the experiences of people with ADHD to those with physical health difficulties may not be suitable and it is suggested that future exploration of transition processes be conducted on a condition by condition basis. This recommendation is made on the basis that adult ADHD services are not fully integrated into AMHS in the UK and therefore may not be comparable with physical health conditions which have longstanding adult services and dedicated models to aid transition. Whilst some of our findings support previous research, the identification of the importance of parental involvement often in a practical capacity and emphasis on clinician qualities affecting overall service perception would benefit from further exploration especially in relation to service engagement and the experience of clinicians in AMHS.

## Conclusions

Our study supports previous findings that timely preparation, transition planning, periods of joint working and consistency in key workers promote a positive experience of transition. Clinicians who are attentive, flexible and go ‘the extra mile’ for their patient foster positivity in young people with ADHD and their families. Our study specifically highlighted the importance of continued support by parents, which is necessary for young people with ADHD during transition and may be potentially necessary during their time with adult services. Furthermore, understanding young people’s experiences of adult services is invaluable and may help to shape future adult services and promote the development of care models for this client group. We hope that the themes identified in this study provide a springboard for further research and exploration.

### Ethical approval

The Derby Research Ethics Committee gave permission for this study. The study gained R&D approval from the Trent CLRN.

## Competing interests

The authors declare that they have no competing interests.

## Authors’ contributions

KDS and VM conducted the qualitative interviews. KDS, VM and CH conducted the coding and thematic analysis of the transcripts, supervised by LR and CH (PI on the project). All authors contributed to the interpretation of the data and the study write-up. KDS drafted the manuscript and VM, CH, LR, KS and CH revised it critically for important intellectual content. All authors read and approved the final manuscript.

## Pre-publication history

The pre-publication history for this paper can be accessed here:

http://www.biomedcentral.com/1471-244X/13/74/prepub
